# 

**Published:** 1871-02

**Authors:** 


### EDITORIAL DEPARTMENT.

*Southern Dental Association*—The Third Annual Meeting of this Association, will be held in Charleston, S. C., on the second Wednesday in April next, and promises to be a highly interesting one to the profession.

While a cordial invitation is extended to all members throughout the country, it is to be hoped that the number, of delegates from the South will be larger than ever before, as this Association is intended more especially for the benefit of Sonthern dentists

The following are the officers for the present year:

Dr. James S. Knapp, *President;* Dr. F. Y. Clark, 1*st Vice-Pres.;* Dr. E. A. Herman, 2*d Vice-Pres.;* Dr. L. E. Edmunson, 3*d Vice-Pres.;* Dr. W. N. Morrison, *Corresponding Sec'y;* Dr. Jno. G. Angell, *Recording Secretary;* Dr. W. G. Redman, *Treasurer.*

*Executive Committee*, Drs. J. B. Patrick, J. Taft, A. C. Ford, W. S. Chandler, and Sam'l Rambo. *Committee on Membership*, Drs. W. G. Redman, A. F. McLain and F. Y. Clark. *Committee on Publication*, Drs. A.F. McLain, C. L. Thurber and F.H Knapp. *Committee on Dental Education*, Drs. J. P. H. Brown, F. J. S. Gorgas, and Wm. Reynolds. *Committee on Physiology and Surgery*, Drs. J, Taft, Wm. H. Morgan and W. T. Arrington. *Committee on Histology and Microscopy*, Drs. S. P. Cutler, W. N. Morrison and Jno. G. Angell. *Committee on Dental Chemistry*, Drs. J. G. McAuley, W. H. Burr, and E. M. Allen. *Committee on Dental Therapeutics*, Drs. F. Y. Clark, G. J. Freidrichs and H. Marshall. *Committee on Operative Dentistry*, Drs. W. H. Morgan, R. N. Laurence, and W. H. Shadoan. *Committee on Mechanical Dentistry*, Drs. C. E. Kells, J. R. Walker, and H. Laurence. *Committee on Dental Literature*, Drs. F. J. S. Gorgas, J. P. H. Brown, and B. F. Arrington. *Committee on Voluntary Essays*, Drs. W. S. Chandler, L. E. Edmunson and L. G. Holland.

*Baltimore College of Dental Surgery.*—The *Thirty-first* Annual Commencement of this institution, will be held at the Concordia Opera House, on Thursday evening, March 2d, 1871. The Alumni and friends of the College are cordially invited to be present, without waiting for special cards of invitation. The Valedictory address will be delivered by Prof. Thos. E. Bond, M. D.

